# Evolution and expansion of the RUNX2 QA repeat corresponds with the emergence of vertebrate complexity

**DOI:** 10.1038/s42003-020-01501-3

**Published:** 2020-12-15

**Authors:** Axel H. Newton, Andrew J. Pask

**Affiliations:** 1 Biosciences 4, The School of Biosciences, The University of Melbourne, Royal Parade, Parkville, VIC 3052 Australia; 2 Present Address: Anatomy and Developmental Biology, The School of Biomedical Sciences, Monash University, Clayton, VIC 3800 Australia

**Keywords:** Molecular evolution, Evolutionary genetics

## Abstract

Runt-related transcription factor 2 (RUNX2) is critical for the development of the vertebrate bony skeleton. Unlike other RUNX family members, RUNX2 possesses a variable poly-glutamine, poly-alanine (QA) repeat domain. Natural variation within this repeat is able to alter the transactivation potential of RUNX2, acting as an evolutionary ‘tuning knob’ suggested to influence mammalian skull shape. However, the broader role of the RUNX2 QA repeat throughout vertebrate evolution is unknown. In this perspective, we examine the role of the RUNX2 QA repeat during skeletal development and discuss how its emergence and expansion may have facilitated the evolution of morphological novelty in vertebrates.

## Abstract (web summary)

In this Perspective, Axel Newton and Andrew Pask examine the role of the Runt-related transcription factor 2 (RUNX2) QA repeat during skeletal development and discuss how its emergence and expansion may have facilitated the evolution of morphological novelty in vertebrates.

## Introduction

Runt-related (RUNX) proteins are a conserved family of DNA-binding transcription factors^1^ that play critical roles during development^2^. RUNX transcription factors are characterized by their conserved Runt domain^3^, first identified through sequence homology to Drosophila *runt*, a pair-rule segmentation gene with multiple developmental roles during embryogenesis^4^. Vertebrate RUNX transcription factors form heterodimeric complexes with core-binding factor-beta (CBFβ) to allosterically enhance DNA binding and promote transcription of their downstream targets^5^. RUNX proteins are fundamental in many developmental processes^2^ such as the regulation of cell cycle kinetics and proliferation^6,7^ and driving cell fate specification and cellular differentiation^8^. RUNX family members are necessary for development as loss-of-function mutations cause embryonic lethality^2^. In addition, the dysregulation of RUNX family members is associated with developmental disorders and cancer^9^.

## Evolution of the RUNX family of transcription factors

**Fig. 1:**
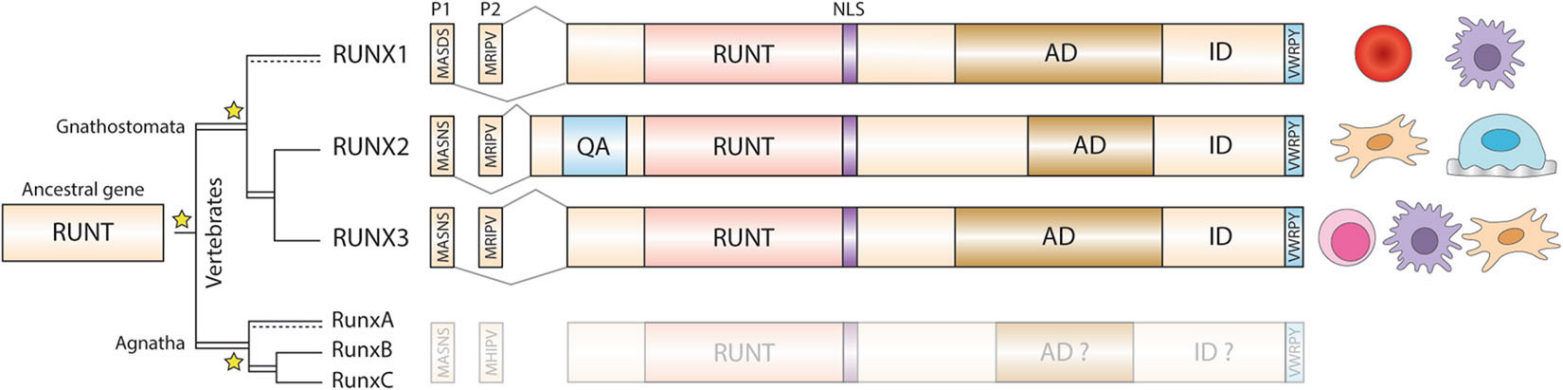
Evolution and structure of the RUNX family of transcription factors. The ancestral metazoan *RUNT* gene locus underwent two independent rounds of duplication (stars, double lines) near the base of the vertebrate tree to derive the three modern RUNX paralogs in gnathostomes and cyclostomes. Each modern RUNX locus contains a conserved dual P1/P2 promoter, and overall structural homology sharing the RUNT DNA-binding domain, activation (AD) and inhibition (ID) domains, nuclear localization signal (NLS), and C-terminus VWRPY motif. Each member has diverged unique tissue specificity, with RUNX1 controlling hematopoiesis, RUNX2 regulating osteogenesis, and RUNX3 playing supporting roles in chondrogenesis and tumor suppression.

RUNT/X genes are found broadly throughout Metazoans^10^. Vertebrates have evolved three paralogs, *RUNX1*, *RUNX2*, and *RUNX3*, suggested to have arisen through two independent duplications of the ancestral *RUNT* gene locus near the base of the vertebrate tree (Fig. 1)^1,11^. Modern RUNX paralogs have maintained strong structural homology and conserved protein domains, and are expressed in two main isoforms from a proximal (P2) or distal (P1) promoter (Fig. 1)^1,2,12^. While RUNX members play important and overlapping functions in many developmental processes (reviewed in ref. ^2^), they each have tissue-specific expression patterns indicating some exclusive roles^2,8,13^. For example, RUNX1 (AML1/CBFA2) is regarded as a master transcription factor regulating hematopoiesis and blood cell development^14,15^. RUNX3 plays an important role in inflammation and tumor suppression^16,17^, while RUNX2 (CBFA1) has evolved a unique role in bone development and is regarded as the master regulator of osteogenesis^18,19^. Unlike other RUNT/X family members, an additional poly-glutamine (Q) (polyQ), poly-alanine (A) (polyA) tandem (QA) repeat domain has evolved within RUNX2 (Fig. 1)^20^, which was found to play a role in protein transactivation^21–25^. Moreover, the RUNX2 QA repeat displays large length variation between species, which is often correlated with skull shape in mammals. RUNX2 QA repeat variation has been suggested to subtly impact its molecular function, leading to increased morphological variation within a population on which selection can act. Genes that play fundamental developmental roles and are prone to variation have been labeled as “evolutionary tuning knobs”^26^, where length variation within these can subtly alter their protein function. However, little is known about the precise role of the RUNX2 QA repeat during skeletal development, nor how and when it emerged during the evolution of vertebrates. In this study, we examine the roles of the RUNX2 QA repeat during osteogenesis and lend perspectives to how changes to its composition may have facilitated the evolution of the vertebrate skeleton. Furthermore, we perform a phylogenetic analysis of the RUNX2 QA repeat across the major vertebrate radiations and discuss how the emergence and expansion of the novel QA domain may have helped to shape vertebrate diversity.

## RUNX2 and osteogenic differentiation

RUNX2 plays an essential role during skeletal development, which we briefly summarize below (for detailed reviews see refs. ^5,27,28^). The two main *RUNX2* isoforms (type I and II) are primarily expressed in developing bone and surrounding supporting tissue^29–34^. RUNX2 plays reciprocal roles during osteogenesis, regulating the commitment of undifferentiated mesenchyme towards an osteogenic fate through activation of the osteogenic gene expression network^20,34^, and in regulating cell cycle kinetics to determine the proliferation rate in osteogenic cell lineages^7,35–38^. RUNX2 initiates the osteogenic differentiation pathway through the activation of other transcriptional regulators, namely the zinc-finger transcription factor *Sp7* (Osterix/OSX), to drive precursor cells towards a mature osteogenic cell fate^39,40^. Together, RUNX2 and OSX upregulate collagens (e.g., *Col1a1, Col1a2*) and osteogenic genes, including Osteocalcin (*Bglap*) and Osteopontin (*Spp1*) for osteoblast maturation, terminal differentiation, and formation of a mineralized, calcified matrix^5,41^. RUNX2 regulates bone formation through two distinct processes: endochondral ossification or intramembranous ossification. Endochondral ossification (e.g., development of the long bones of the limbs) occurs when mesenchymal stem cells differentiate into chondrocytes through the activation of the transcription factor SOX9^42^. Chondrocytes grow and divide to form a cartilage anlage, which later matures and is converted to bone through the activation of RUNX2^43,44^. Intramembranous ossification (e.g., development of the major facial bones and lateral end of the clavicles) occurs when RUNX2 directly regulates the differentiation of mesenchymal stem cells into osteoblasts^40,45,46^. Here, RUNX2 regulates the rate of mesenchymal cell proliferation and differentiation^6,47^, directly influencing the rate of growth and formation of bone.

The necessary role of RUNX2 during skeletal development is seen in *Runx2*-deficient mice, which fail to develop membranous and endochondral bone and have an atrophied cartilaginous skeleton^18^. Physiological development of the skeleton is RUNX2 dose-dependent, where alterations to RUNX2 expression levels impact the size and shape of bone^48^. For example, global RUNX2 haploinsufficiency produces the congenital disorder cleidocranial dysplasia (CCD)^19^. CCD patients display membranous bone abnormalities such as missing or reduced clavicles and craniofacial defects, including a prominent forehead, wide and short skull (brachycephaly), wide eyes (hypertelorism), flat nose, small upper jaw (maxillary hypoplasia), and incomplete closure of the cranial sutures^19^. Similarly, knockdown or overexpression of RUNX2 in targeted regions can specifically influence the rate of bone growth, without global effects^49,50^. As such, the temporospatial expression and activation of RUNX2 during skeletal development can ultimately determine the size and shape of individual membranous bones^49,51^. This demonstrates that not only is RUNX2 an essential regulator of osteogenesis and development of the cranial and postcranial skeleton^20,52^, but alterations to its expression, regulation, or activation during development may generate novel skeletal variation^49^.

## Protein coding repeats and the RUNX2 QA domain

Of the three RUNX family members, RUNX2 uniquely contains a polyQ, polyA tandem repeat domain. Tandem repeat domains can facilitate a diverse range of biological roles^53–55^, including gene transactivation^21^, intracellular protein translocation^25,56,57^, protein–protein, protein–DNA, and protein-RNA interactions^55,58^. Tandem repeat domains possess high length variability due to their tendency for mutations during DNA replication. High purity repeats with uninterrupted codon composition (e.g., CAG CAG CAG CAG : QQQQ) are more likely to stutter during replication causing homopolymeric expansions or contractions. On the other hand, low purity repeats with variable codon usage (e.g., CAG CA**A** CAG CA**A** : QQQQ) are generally more stable and less prone to mutation^59^. Repeat slippage mutations occur more frequently than point mutations^60^ and are able to rapidly generate multiallelic variation^55^ without deleterious consequences such as frameshifts or premature stop codons. As such, alleles with novel repeat lengths can facilitate variation within a population and those that have beneficial functions can become fixed by selection^61^. Therefore, protein coding repeats have been referred to as evolutionary “tuning knobs”^26,60^, acting to rapidly generate beneficial genetic variation, independent of longer-term evolutionary epistasis^61–64^. (Both nucleotide sequences code for the same homopolymeric amino acid sequence, consisting of 4 glutamines (QQQQ). Bold letters denote how changes to the codon nucelotide sequence do not affect the amino acid sequence, but do change the nucleotide composition that codons are alternated rather than being repetitive).

Tandem repeat domains are present in a wide range of developmental genes and transcription factors^61,65,66^ and possess substantial intra- and inter-species-specific variation^67^. However, the biological roles of repeat expansions and contractions are not entirely clear, and often only described in the context of disease. For example, polyQ repeats in coding genes can influence protein–protein interactions with transcriptional co-activators to regulate gene expression^68,69^ in a length-dependent manner^56^. However, long repeats can become unstable, forming toxic β-sheet aggregates. This is seen in Huntington’s disease, caused by large unstable polyQ expansions in the HTT (*Huntingtin*) gene^70^. The role of polyA repeats is less understood, although they are suggested to facilitate protein translocation between subcellular compartments^25,71,72^. For example, expanded polyA repeats in RUNX2 cause cytoplasmic aggregation, decreasing protein availability in the nucleus^25^. Humans and mice with homozygous polyA expansions in the homeobox gene *Hoxd13* develop synpolydactyly; however, this phenotype is absent in heterozygous individuals^73,74^. Yet, while individual polyQ or polyA tracts are common in many proteins^75^, tandem polyQA repeat domains are exceedingly rare and their precise roles remain poorly understood. The best example is observed in RUNX2, which has been implicated in the fine-tuning of protein transactivation^55^.

**Fig. 2:**
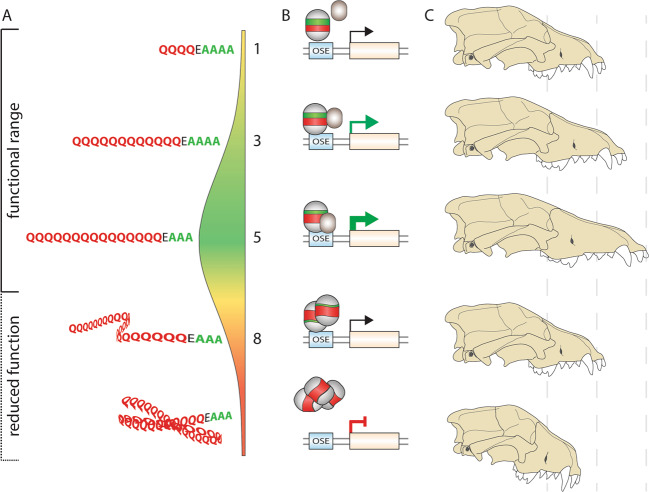
RUNX2 QA repeat length determines protein transactivation. Schematic of RUNX2 QA repeat mode of action. **a** QA repeat lengths form a “goldilocks” range that determines function. Short repeats are less functional than medium length repeats, while expanded repeats form protein aggregates, reducing function. **b** Hypothetical mechanism of action where increasing repeat length promotes interactions with transcriptional co-factors, increasing gene expression (arrows) before hitting a critical threshold inhibiting activity. **c** QA repeat-driven changes to protein transactivation and downstream gene expression is suggested to fine-tune craniofacial length in several groups of mammals and can cause disease when in excess. OSE osteocalcin-specific element that occurs in osteogenic gene promotors.

The mammalian RUNX2 polyQ and polyA repeats sit within immediate proximity of each other, separated by a single glutamic acid (E) spacer. The polyQ and polyA domains each form individual α-helix coiled coils, which interact to form a super-coiled secondary structure^25,57^. In addition to variation in overall repeat length, the ratio of glutamine-to-alanine residues (Q:A ratio) in RUNX2 can also alter the stability of the coiled coil^25^, altering the affinity for protein–protein interactions and thus gene transactivation^66^. RUNX2 QA repeat length polymorphisms within a functional range influence its activity, providing a length-dependent mechanism to control gene transactivation. This has been empirically determined in vitro where RUNX2 QA alleles with increasing Q repeats promote transactivation and gene expression^22,24^. However, Q or A repeats outside the functional range become unstable, lose their transactivation potential, and form aggregates (Fig. 2a, b)^21,23,25,76^. Targeted deletion of the RUNX2 QA domain (*Runx2*ΔQA) results in a significant reduction in gene transactivation compared to the wild-type allele in vitro^21^. Also, conditional knock-in of wild-type *Runx2* and the *Runx2*ΔQA allele in mouse hypertrophic chondrocytes (terminal cartilage) induced osteogenic differentiation and bone mineralization in vivo, although overall this level was reduced in ΔQA mice^77,78^. Together, these examples demonstrate that while the RUNX2 QA repeat influences osteogenic gene transactivation and the rate of osteogenesis, it is not essential for osteoblast differentiation and bone development. Here, the polyQ domain regulates transactivation levels, while the polyA may support translocation and protein stabilization, although these may be further controlled by variable Q:A ratios.

Importantly, alterations to the QA ratio can influence the global development of the skeleton and impact morphology. Human RUNX2 variants have revealed that small QA length polymorphisms can subtly, but significantly alter bone mineral density^23,24,79^, while larger expansions cause subtle CCD-like craniofacial variation and brachydactyly^76,80^. These findings further demonstrate how variation of the QA domain can alter RUNX2 activity and osteogenic gene transactivation, before reaching a critical threshold and ultimately becoming unstable and causing disease (Fig. 2a, b).

## The mammalian RUNX2 QA repeat and craniofacial evolution

A potential role for the RUNX2 QA repeat in morphological evolution was first identified between breeds of domestic dogs (*Canis lupus familiaris*). Modern dog breeds display a wide range of skeletal and craniofacial variation as a result of their strong selective breeding (artificial selection). Fondon and Garner^61^ examined protein repeat length polymorphisms in 36 developmental genes across 92 dog breeds. Overall, dogs possessed elevated repeat purity (see also ref. ^62^) and many repeat length polymorphisms in key transcription factors^61^. Of these, large variability was detected in RUNX2 QA repeat length and ratio, which was found to be positively correlated with facial shape, namely the length (*r*^2^ = 0.63) and angle of facial bones (klinorhynchy; *r*^2^ = 0.51) relative to skull length, between breeds^61^. Lower QA ratios occurred in breeds with short-faced morphologies, and higher QA ratios in long-faced breeds. Experimental evidence has shown that RUNX2 QA repeat variation can alter its transactivation potential. This may impact the timing or rate of osteogenesis in the developing facial bones, with subtle QA variation influencing craniofacial shape and size (Fig. 2c). This supports the “tuning knob” hypothesis^26,60^ where RUNX2 QA repeat polymorphisms can impact its function, which in turn, may produce morphological variation that can be subsequently fixed through selection. However, it is important to note that dog breeds represent a single species under extreme artificial selection, and mechanisms that enable rapid variation (such as coding repeat domains) to promote trait evolvability will be favored under such extreme selective pressures. Thus, the question remained whether variation in the RUNX2 QA domain could explain craniofacial variation in naturally evolving species.

Correlations between RUNX2 QA repeat length/ratio and facial shape metrics have been previously investigated throughout several groups of naturally evolving mammals with diverse craniofacial morphologies. These relationships have been examined within marsupial (metatherian)^81^ and placental (eutherian) mammals^82^, including Primates^83,84^, Carnivora^22^, and Chiroptera (family Phyllostomidae)^84^. For each examined species, the RUNX2 polyQ and polyA repeats were determined, and the QA ratio was calculated. These data were then compared to specific facial shape metrics, namely the length or width of the membranous facial skeleton. Species QA ratios vs. facial “shape” ratios were individually plotted, and correlations were determined for each group.

Marsupials possess diverse facial shapes, but showed little to no variation in their RUNX2 QA repeat at any phylogenetic level, despite possessing high overall repeat purity^81^. This was suggested to be in response to the functional constraints placed on skeletal development in the highly altricial young of marsupials, which require accelerated bone development to support their unique mode of reproduction (see refs. ^85–87^). Therefore, the marsupial RUNX2 repeat was hypothesized to be under strong purifying selection^81^. However, in contrast, RUNX2 QA repeat ratios were found to be significantly correlated with the facial shape within all examined eutherian orders. Primates possess a small range of RUNX2 repeat ratios (Q:A = 1.1–1.7), which were positively correlated with facial length (relative to total skull length; *r*^2^ = 0.23, *p* < 0.005)^83^. Carnivorans possess a wide range of QA ratios (1.5–5.33), which were positively correlated with facial length (relative to total skull length, *r*^2^ = 0.24, *p* = 0.006)^22^ and chiropteran phyllostomids possess a small range of QA ratios (1.2–2.33), which were negatively correlated with palate length (*r*^2^ = −0.51, *p* = 0.003) and positively correlated with palate width (*r*^2^ = 0.55, *p* = 0.001), relative to the geometric mean of overall skull shape^84^. Although when RUNX2 repeat and facial length were compared more broadly across eutherian superorders (Laurasatheria, Xenartha, Eurachontoglires, and Afrotheria), correlations were absent (*r*^2^ = <0.1, n.s.)^82^, suggesting that this relationship exists in epistasis with other factors controlling bone development. However, across smaller evolutionary distances, QA repeat variation may facilitate adaptive morphological evolution, although the definitive role of RUNX2 QA repeat during craniofacial morphogenesis is yet to be determined.

Together, these data lend support to the hypothesis that RUNX2 QA repeat variation may function as an evolutionary “tuning knob”^26,60^ generating rapid, adaptable variation over short evolutionary distances. However, amino acid repeats are highly volatile sequences that can become unstable, causing detrimental cellular effects^84^. As such, over larger evolutionary distances, these inherent risks are likely compensated for by other osteogenic gene regulatory changes^88,89^, illustrated by the absence of RUNX2 repeat length vs. facial shape correlations between eutherian orders. Nevertheless, variability within the RUNX2 QA repeat appears to have played important roles in facilitating the evolution of the mammalian facial skeleton^22,81–84^. However, little is known about the QA repeat in other vertebrate clades, when it appeared during vertebrate evolution, nor the roles it may have played in the evolution of the vertebrate skeleton. For the remainder of this perspective, we examine when during vertebrate history the RUNX2 QA repeat emerged and how changes in its composition and structure correspond with the evolution of the major vertebrate radiations.

## Evolution of the RUNX2 QA repeat across vertebrate history

The modern RUNX paralogs arose early during vertebrate evolution through whole-genome duplication events. Approximately 50 mya during the late Cambrian, the ancestral vertebrate genome underwent its first duplication, prior to the divergence of the agnathan and gnathostome lineages (Fig. 1)^90–92^. Around 50 mya later in the Ordovician, the agnathan and gnathostome lineages underwent additional, independent whole-genome duplications (Fig. 1)^90–92^. Signatures of these duplication events are observed through comparative sequence analysis of *RUNX* genes in chordates, where non-vertebrate cephalochordates (amphioxus) and tunicates possess a single *RUNT/X* gene^1^, while all extant lineages of vertebrates possess three *RUNX* paralogs^93^. However, the three *RUNX* genes in the basal cyclostome (agnathan) lamprey and hagfish (*RunxA*, *RunxB*, and *RunxC*) are not true one-to-one orthologs with the gnathostome *RUNX* genes (*RUNX1*, *RUNX2*, and *RUNX3*)^11^ supporting independent gene duplication events^92^ (Fig. 1). In addition, phylogenetic comparisons of modern RUNX paralogs reveal greater similarity between the *RUNX2* and *RUNX3* gene loci, suggesting that they diverged after the initial vertebrate whole-genome duplication^1,92^. Therefore, *RUNX2* evolved in the last common ancestor of gnathostomes ~450 mya^1,11,92^.

It remained unclear when the QA repeat evolved after the divergence of the gnathostome RUNX2 paralog, and what role it may have played in the evolution of the vertebrate skeleton. As such, we examined the N terminus of RUNX2, containing the QA repeat and flanking sequences, from 409 vertebrate species covering all extant lineages (from publicly available sequences on GenBank, Ensembl, Short-read Archive (SRA), and published observations; Supplementary Methods). The RUNX2 QA repeat domain was defined as the intermittent region between a conserved, N-MSDVS and C-VPRLR motif, at the N terminus of the RUNX2 protein, immediately upstream of the RUNT domain. We defined sequences that lacked an obvious polyQ or polyA, but contained flanking motifs resembling MSDVS and VRPLR as the proto-QA domain. The presence of mostly conserved MSDVS and VPRLR motifs with a short or interrupted polyQ/polyA domain was defined as a primitive QA domain, while an uninterrupted and variable length RUNX2 QA repeat was defined as the variable polyQA domain. We then compared the evolution and expansion of the RUNX2 QA repeat domain across a consensus vertebrate phylogeny (Supplementary Data 1) with median divergence times^94^.

**Fig. 3:**
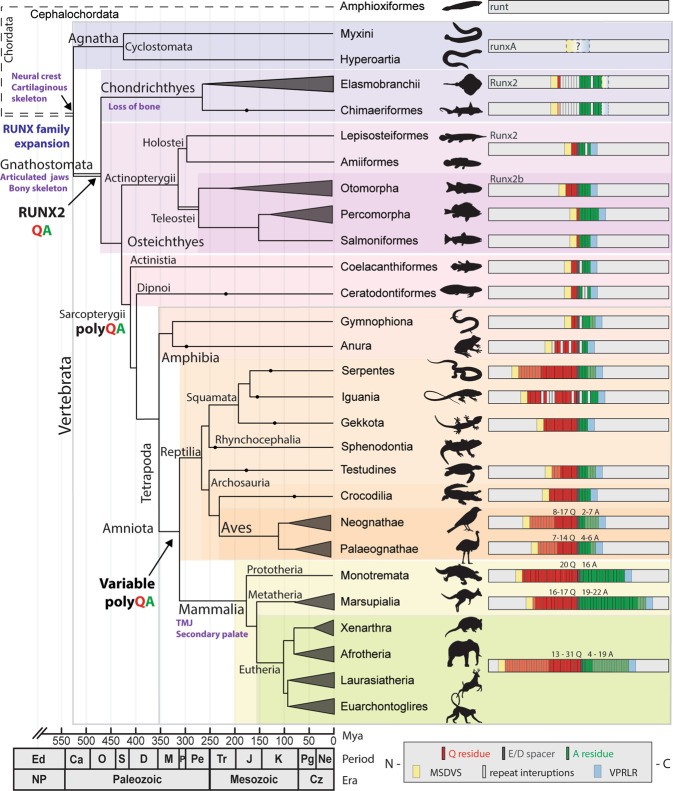
RUNX2 QA repeat evolution throughout vertebrates. Simplified phylogeny showing the evolution of the RUNX2 QA repeat during vertebrate history. Evolutionary relationships between major vertebrate radiations are shown with median divergence times. Representative species of each group are shown by silhouettes, and repeat structures with Q, spacer, and A residues are shown for each group on the right. The QA repeat emerged in the last common ancestor of gnathostomes and can be observed in all subsequent extant lineages. The repeat continued to expand throughout early tetrapods and amniotes, before reaching its long and variable modern condition in eutherian mammals <100 million years ago. Taxa silhouettes were created from images under a CC BY 4.0 open license.

The divergence of the RUNX2 paralog occurred during the Ordovician and may have facilitated the evolution of complex vertebrates (Fig. 3). The duplication and divergence of RUNX2 (and RUNX3) occurred after the appearance of cartilage^95^, but before the evolution of the bony skeleton, suggesting it may have been co-opted to establish the primitive skeletal gene regulatory network^96–98^. We were unable to detect the presence of the proto-QA repeat domain in the non-vertebrate, cephalochordate or tunicate runt genes (Supplementary Fig. S1). In vertebrates, we failed to detect a proto-QA domain in the jawless cyclostome RunxA, B, or C, although we did identify some similar features such as sporadic polyQ/H tract in lamprey RunxA (Supplementary Figs. S1, 3). However, we detected the presence of at least the RUNX2 proto-QA domain in all available sequences from extant lineages of gnathostomes. The jawed, but cartilaginous, Chondrichthyes (sharks and rays) possessed a small domain with discernable flanking motifs and a short polyA, but lacked a polyQ (Fig. 3, Supplementary Fig. S1, and Supplementary Data 2).

We also detected a RUNX2 primitive QA domain throughout all sampled lineages of Osteichthyes bony fish, namely the actinopterygian (ray-finned) Holostei and Teleostei radiations, and sarcopterygian (lobe-finned) Coelacanth (subclass Actinistia) and air-breathing lungfish (subclass Dipnoi)^94^ (Fig. 3, Supplementary Fig. S1, and Supplementary Data 2). The osteichthyan primitive QA repeat was observed as a small domain with conserved flanking motifs and short polyQ and polyA tracts (Fig. 3). Interestingly, Teleost fish underwent an additional lineage-specific whole-genome duplication^99^, producing two Runx2 copies (*Runx2a* and *Runx2b*) expressed in skeletal tissues^100^. However, limited sequence data suggest that both orthologs possess the primitive RUNX2 QA repeat, so the influence of this repeat duplication remains unclear. Together, the lack of a QA repeat in cephalochordate/tunicate runt, cyclostome RunxB, and gnathostome RUNX1/3, combined with the presence of a proto-QA domain in extant Chondrichthyes and primitive QA domain in extant Osteichthyes RUNX2, strongly suggests that the RUNX2 QA repeat first evolved after the duplication and divergence of RUNX2, but in the last common ancestor of extant gnathostomes <450 mya.

Curiously, prior to the duplication and divergence of the RUNX2 paralog and evolution of the gnathostomes, several lineages of jawless fish populated the ancient oceans, which possessed “bony” exoskeletal headshields (i.e., the Galeaspida, Pituriaspida, and Osteostraci). However, it is unclear as to whether these early lineages possessed or utilized RUNX2 to develop their ossified headshields due to a lack of extant representatives. Similarly, among the first jawed vertebrates were the extinct Placoderms, known for their large “bony” exoskeletal armor plating^97^. Although it remains unclear whether they possessed RUNX2 and a proto- or primitive QA repeat. The evolution of the Osteichthyan fish marks a major transition in vertebrate evolution, signified by the first emergence of a true bony endoskeleton and skull^96,97,101^. The evolution of the RUNX2 QA domain, coinciding with this major vertebrate transition, may have established a novel molecular function during early ossification and skeletal development, particularly given the known transcriptional activation roles of the polyQ^56,68,69^ and protein translocation roles of the polyA domains^71,72^. Although small, the proto- and primitive QA domains may have established novel protein–protein interactions or affinities for transcriptional co-activators^56,68,69,71,72^ enhancing ossification capacity in the early skeleton. The early emergence of the proto-QA provided a template for the evolutionary expansion of the modern QA domain, which may have acted as a primer for the evolution of morphological complexity in tetrapods.

The emergence of a variable length RUNX2 polyQA repeat occurred ~350 mya during the tetrapod radiation, first observed in the amphibia^94^. Amphibians represent the most basal tetrapod lineage and possess a primitive/variable QA repeat with conserved flanking residues, a distinct spacer residue, and polyQ and polyA domains with interrupting residues, which vary in length between groups. Caecillians (order Gymnophiona) possess a short QA domain (2 Q:3–5 A), while frogs and toads (order Anura) have evolved an expanded polyQ domain (6–10 Q:2 A) with several proline interruptions (Supplementary Data 3 and Fig. 3). The initial expansion of the repeat domain may have required compensatory changes for novel protein functions. For example, proline interruptions in both polyQ and polyA repeats are found to decrease coiled-coil stabilization, reducing expansion-related protein aggregation^25,57^.

Expansion of the variable RUNX2 QA repeat occurred with the emergence of amniotes ~312 mya, observed in all extant lineages of Reptilia, Aves, and Mammalia^94^. Reptilian clades, such as the Rhynchocephalia (7 Q:6 A), Testudinata (turtles and tortoises; 6–9 Q:3–7 A), and Crocodilia (10 Q:4 A) have a short repeat with minor interspecific length variation (Supplementary Data 4). However, groups such as Squamata (snakes, lizards, and geckos) and Aves (birds) have a large repeat with intraspecific variability. For example, snakes (Serpentes, Squamata) have evolved a long polyQ domain (12–21 Q; QA ratio 2.0–5.25; Supplementary Data 4 and Fig. 3). Similar length expansions and variation were observed in taxa from the Iguania (Squamata), largely in Anolis lizards. Anoles are well known for their extraordinary adaptive radiation^102^ and convergent ecomorphs in cranial shape arising over the past 50 million years^103–106^. Members of the *Anolis* genus showed large variation in repeat length and composition (Supplementary Data 4), having a short polyA repeat but expanded and variable polyQ domain (10–27 Q:4–6 A) with several histidine, alanine, proline, and serine interruptions. As mentioned previously, these may have evolved to compensate for the rapid and hyper-expansion of their repeat structure to reduce protein aggregation and stabilize function^25,57^. Interestingly, the adaptive diversification of the anole skull may have been facilitated by RUNX2 QA variation to enable the rapid evolution of disparate and convergent ecomorphs. As such, the disparity in *Anolis* RUNX2 QA repeat length and ratio provides a unique opportunity to examine whether QA repeat variation facilitates the adaptive evolution of facial shape in a naturally evolving family, acting as a natural counterpoint to observations in domestic dogs^61^.

Birds (Aves) represent a relatively recent vertebrate evolutionary radiation, diverging from their therapod dinosaurian ancestors ~160 mya, with the modern avian crown group appearing ~111 mya. Modern birds have since diversified into over 10,000 different species^94,102^, displaying a broad spectrum of sizes and body forms in response to their unique ecologies. This is reflected by many specific skeletal adaptations in response to their locomotor demands (i.e., powered flight, swimming, gliding, or terrestrial bipedalism) and remarkable diversity in beak shape reflective of their specialized diets^107^. Interestingly, birds possess minor RUNX2 QA repeat length variation within sampled Neognathae and Palaeognathae orders, although some groups display large inter-order variation. For example, sampled Tinamiformes possess low Q:A ratios (*n* = 3; Q:A ≤ 1.80), while sampled Galliformes possess conserved, high QA repeat ratios (*n* = 8; Q:A 6.0; Supplementary Data 5). This high intra-order conservation of QA repeat length is in contrast to mammals, which show large intra-order variation suggested to facilitate facial shape evolution^22,82–84^. Instead, the ancestors of modern bird groups may have utilized repeat length variation to promote lineage-specific radiations and adaptive beak evolution^107–109^, subsequently preserved within extant species. An unpublished study examining correlations between RUNX2 QA repeat composition and beak length in shorebirds (Charadriiformes) found that, in contrast to our hypothesis, that this order possesses highly variable QA ratios (1.86–4.25) that are weakly correlated with beak length (*R*^2^ = 0.13)^110^. However, RUNX2 QA repeat sequences for these taxa are not publicly available, so this result could not be verified. Additional correlative examinations of RUNX2 QA repeat vs. beak shape will help define the role of RUNX2 in avian morphological diversification.

Mammals diverged from other amniotes ~177 million years^94,111^ and have since evolved a remarkable array of adaptations in response to terrestrial, arboreal, aerial, subterranean, and aquatic environments. Mammals are characterized by several unique craniofacial traits, such as the secondary palate and temporo-mandibular joint, which have established novel feeding ecologies through jaw articulation^101,112–115^. These novel morphological characteristics coincide with the longest RUNX2 QA repeat of all vertebrate lineages examined. Extant monotremes (*n* = 2, 16–20 Q:6–19 A) and marsupials (*n* = 26, 16–24 Q:19–22 A) possess some minor RUNX2 repeat variation, while eutherian mammals (*n* = 162, 7–31 Q:4–19 A) display extreme repeat length variation across extant lineages (Supplementary Data 6 and Fig. 3). For example, the naked mole-rat (*Heterocephalus glaber*) has the largest ratio (31 Q:5 A, QA ratio = 6.2), while the Baiji, or Yangtze River dolphin (*Lipotes vexillifer*) has the shortest ratio (7 Q:16 A; QA ratio = 0.44; Supplementary Data 6). Eutherian mammals diverged from the marsupials and monotremes, ~160 and ~177 mya, respectively, and radiated into four superorders ~105 mya^94,111^. As such, the extreme RUNX2 QA repeat variation observed between eutherian mammals is a recent phenomenon occurring within the past ~100 mya.

## Future directions

We highlight several independent studies with consistently recovered correlations between RUNX2 QA repeat variability and facial length morphology at multiple taxonomic levels^22,82–84^. However, additional examinations in other groups of naturally evolving vertebrates will further support the role of the RUNX2 QA repeat variation underlying morphological evolution. For example, the previously mentioned Anoles represent a natural case study by which RUNX2 repeat evolution may influence adaptive convergence of cranial ecomorphs^107^ and studies in birds will elucidate whether RUNX2 QA repeat variation promotes adaptive changes in beak shape diversity^107^. In addition, leporid rabbits and hares (order Lagomorpha) are a relatively recent (~20 mya) evolutionary radiation that display substantial craniofacial length and angle variation in response to different ecologies^116,117^. However, how RUNX2 QA repeat variation corresponds with the natural craniofacial variation in this group remains unknown.

The reported within-group correlations, combined with several lines of empirical evidence for variable polyQ and polyA repeats altering RUNX2 transactivation, suggest that the RUNX2 QA repeat is a putative mechanism that can produce morphological variation that selection can act upon. However, it is important to note that while these anecdotal correlations and empirical evidence exist, the direct influence of RUNX2 QA repeat variation on skeletal morphogenesis is still yet to be determined. Experimental approaches utilizing genome editing in a range of model vertebrates^118^ will unequivocally reveal the role of RUNX2 QA repeat variation on the formation of the vertebrate skeleton. Particularly, replacement of the endogenous RUNX2 QA domain with varying length QA polymorphisms will allow precise quantification of its contribution to bone development and morphological evolution.

## Concluding remarks

In this perspective, we examined the origin and emergence of the RUNX2 QA repeat across the evolution of vertebrates and drew comparisons with empirical studies to determine how this may have shaped vertebrate diversity. This is the first study to assess broad taxonomic sampling with deep evolutionary coverage to analyze sequence variation in the RUNX2 repeat domain. Through this investigation, we have uncovered several fascinating concordances of the emergence and expansion of the RUNX2 QA repeat domain with the major vertebrate transitions. The duplication and divergence of the RUNX2 paralog from the ancestral *RUNT* gene ~450 mya may have primed the development of a bone-specific program, establishing the evolution of the vertebrate bony skeleton. The skeleton supported the emergence of morphological novelty across vertebrates, acting as a scaffold for unique adaptations. The internal *RUNX2* protein QA repeat, absent in the structurally homologous *RUNX1* and *RUNX3*, has provided a novel functional mechanism to fine-tune osteogenesis through enhanced protein–protein interactions and gene transactivation. Therefore, the evolution of the RUNX2 QA repeat has likely played a critical role in shaping the wide range of diversity seen across vertebrates.

Since first appearing in the ancestor of gnathostomes, the QA repeat has sequentially emerged, evolved, and expanded through the divergence of vertebrates, reaching its highly variable configuration with the evolution of amniotes ~312 mya. However, the broad repeat variability observed in eutherian mammals is a recent evolutionary event occurring within the past ~100 million years. The gradual evolution of the internal QA repeat throughout vertebrates may have promoted an increasing ability to subtly alter the development of the craniofacial skeleton through its direct role in intramembranous ossification. The progressive expansion and stabilization of the QA repeat throughout vertebrates demonstrate that it has been fixed during evolution, emphasizing its important roles. While additional studies are required to define the precise role of the RUNX2 QA repeat during skeletal development, the evolution and emergence of the RUNX2 QA repeat provide an intriguing putative mechanism underlying vertebrate evolution.

## Supplementary information

Supplementary information

Description of Additional Supplementary Files

Supplementary Data 1

Supplementary Data 2

Supplementary Data 3

Supplementary Data 4

Supplementary Data 5

Supplementary Data 6

## Data Availability

All sequence data that support the findings of this study are deposited in the GenBank, SRA, Whole-Genome Shotgun, and TSA repositories with accession codes listed in the Supplementary files.
