# Author Correction: Evolution and expansion of the RUNX2 QA repeat corresponds with the emergence of vertebrate complexity

**DOI:** 10.1038/s42003-021-01687-0

**Published:** 2021-01-25

**Authors:** Axel H. Newton, Andrew J. Pask

**Affiliations:** 1 Biosciences 4, The School of Biosciences, The University of Melbourne, Royal Parade, Parkville, VIC 3052 Australia; 2 Anatomy and Developmental Biology, The School of Biomedical Sciences, Monash University, Clayton, VIC 3800 Australia

**Keywords:** Molecular evolution, Evolutionary genetics

Correction to: *Communications Biology* 10.1038/s42003-020-01501-3, published online 15 December 2020.

In the original published version of the Article, the string of glutamines (QQQQ) in the Results section text “e.g., CAG CA**A** CAG CA**A** : **QQQQ**” was incorrectly bolded and underlined, changing the scientific interpretation of the text. The error has been corrected in the PDF and HTML versions of the Article.

