# Supplementary material for: Evolution and expansion of the RUNX2 QA repeat corresponds with the emergence of vertebrate complexity
Source: Commun Biol. 2020 Dec 15;3:771. doi: 10.1038/s42003-020-01501-3 (PMC7738678; doi:10.1038/s42003-020-01501-3)
Supplement: Supplementary file 1 — Supplementary information [file 42003_2020_1501_MOESM1_ESM.pdf]

# Evolution and expansion of the RUNX2 QA repeat corresponds with the emergence of vertebrate complexity

## Supplementary Material

***Communications Biology***

Axel H Newton[1,2] and Andrew J Pask[1]

1. Biosciences 4, The School of Biosciences, The University of Melbourne, Royal Parade, Parkville, Victoria, 3052

2. Anatomy and Developmental Biology, The School of Biomedical Sciences, Monash University, Clayton, Victoria, 3800 (current address)

**Supplementary Methods**

In this study we extracted annotated and unannotated RUNX2 nucleotide and protein sequences from published studies and publically available, curated sequence databases. Annotated sequences were extracted from the Genbank nucleotide/ protein database. Unannotated sequences were identified using BLAST+ (v2.10.1) from transcript databases: Sequence Read Archive (SRA), Transcriptome Shotgun Assembly (TSA) Sequence Database and Whole Genome Shotgun (WGS) database. Sequence accession numbers and sources can be found in Supplementary data 2-6.

Annotated RUNX2 nucleotide/protein sequences were extracted for all available species of each vertebrate clade. Nucelotide sequences were translated into protein using the ExPASy translate tool [1] and the QA repeat domain determined through its location at the N-terminus of the protein and conserved flanking residues homologous to N-terminus MASNS and C-terminus VPRLR. Next, we chose high quality RUNX2 nucleotide sequences from a species representative of each vertebrate clade and trimmed to cover the RUNX2 QA repeat and 50bp of flanking sequence. Representative sequences were used to discover unannotated species-specific sequences from TSA, WGS and SRA databases using discontigous megaBLAST. Searches were performed using NCBI taxonomy IDs to specifically identify RUNX2 QA repeat sequences from species covering each major vertebrate clade. Additional searches within orders were performed to increase sampling where possible. SRA datasets were BLAST searched if they contained >1 billion bases of genomic data for a given species. All nucleotide sequences were converted to protein with ExPASy translate tool and listed in Supplementary data 2-6. Flanking motifs are bolded to denote the beginning and end of the QA repeat domain.

A simplified vertebrate phylogenetic tree was constructed usig PhyloT (v2.0, BioByte) based off NCBI taxonomy, and visualized and exported through the interactive tree of life (iTOL; v5.6.3; BioByte; Supplementary data 1). Branch lengths were calibrated from median divergence time estimates from TimeTree. RUNX2 QA repeats were then mapped onto the simplified phylogeny to visualize the emergence and expansion of the QA repeat throughout vertebrate evolution.

To further examine to emergence, expansion and stablization of the QA domain, we aligned representative N-terminus RUNX2 protein sequences using the Multiple sequence Alignment and NJ / UPGMA phylogeny tool (MAFFT v7.0) [2] from human (*Homo sapiens*), opossum (*Monodelphis domestica*), quail (*Coturnix japonica*), crocodile (*Crocodylus porosus*), gecko (*Gekko japonicus*), caecilian (*Geotrypetes seraphini*), coelacanth (*Latimeria chalumnae*), garfish (*Lepisosteus oculatus*), zebrafish (*Danio rerio*) and ghostshark (*Callorhinchus milii*), representing eutherian and marsupial mammals, birds, archosaurs, reptiles, amphibians, sarcopterygian fish, holeost and teleost bony fish and cartilaginous fish, respectively (Fig. S1). We also included RunxA from the Agnathan lamprey (*Lethenteron camtschaticum*) and hagfish (*Myxine glutinosa)*, and runt from amphioxious (*Branchiostoma floridae*; Fig S1) as an outgroup.

Correlations between RUNX2 repeat length and mammalian facial length ratios were previously examined [3–9].

**Supplementary References**


1. Gasteiger, E. *et al.* ExPASy: the proteomics server for in-depth protein knowledge and analysis. *Nucleic Acids Res.* **31**, 3784–3788 (2003).
2. Katoh, K., Rozewicki, J. & Yamada, K. D. MAFFT online service: multiple sequence alignment, interactive sequence choice and visualization. *Brief. Bioinform.* **20**, 1160–1166 (2017).
3. Newton, A. H., Feigin, C. Y. & Pask, A. J. RUNX2 repeat variation does not drive craniofacial diversity in marsupials. *BMC Evol. Biol.* **17**, 1–9 (2017).
4. Ritzman, T. B. *et al.* Facing the facts: The Runx2 gene is associated with variation in facial morphology in primates. *J. Hum. Evol.* **111**, 139–151 (2017).
5. Sears, K. E., Goswami, A., Flynn, J. J. & Niswander, L. A. The correlated evolution of Runx2 tandem repeats, transcriptional activity, and facial length in Carnivora. *Evol. Dev.*

**9**, 555–565 (2007).

6. Ferraz, T. *et al.* Contrasting patterns of RUNX2 repeat variations are associated with palate shape in phyllostomid bats and New World primates. *Sci. Rep.* **8**, 1–10 (2018).
7. Fondon, J. W. & Garner, H. R. Molecular origins of rapid and continuous morphological evolution. *Proc. Natl. Acad. Sci.* **101**, 18058–18063 (2004).
8. Pointer, M. A. *et al.* RUNX2 tandem repeats and the evolution of facial length in placental mammals. *BMC Evol. Biol.* **12**, 103 (2012).
9. Green, R. M. & Kimball, R. T. Analysis of RUNX2 gene's influence on bill morphology within shore birds. *Thesis* **3737**, (The University of Florida, 2012).

**Supplementary Files**

**Figure S1.** Vertebrate alignment of RUNX2 N terminus containing the QA repeat
Alignment of RUNX2 QA repeat region from representative taxa of all sampled vertebrate lineages (from Figure 3). The proto-QA domain can be distinguished in all vertebrate lineages through homology to the conserved MSDVS and VPRLR motifs flanking the intermittent repeat region. We were unable to identify any signature of the proto-QA domain in either cephalochordate (Brachiostoma floridae) or tunicate runt (not shown).

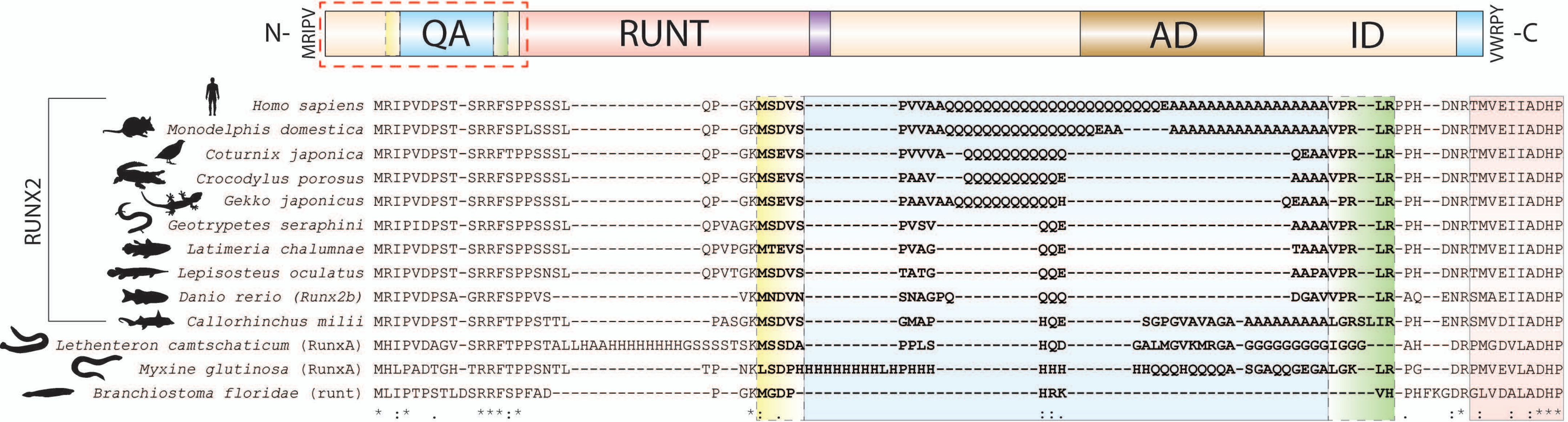
N-
MRIPV
QA
RUNT
AD
ID
VWRPY
-C
RUNX2
Homo sapiens MRIPVDPST-SRRFSPPSSSL--------------QP--GKMSDVS----------PVVAAQQQQQQQQQQQQQQQQQQQQQQEAAAAAAAAAAAAAAAAAVPR--LRPPH--DNRTMVEIIADHP
Monodelphis domestica MRIPVDPST-SRRFSPLSSSL--------------QP--GKMSDVS----------PVVAAQQQQQQQQQQQQQQQQQEAA-----AAAAAAAAAAAAAAAAVPR--LRPPH--DNRTMVEIIADHP
Coturnix japonica MRIPVDPST-SRRFTPPSSSL--------------QP--GKMSEVS----------PVVVA--QQQQQQQQQQ-----------------------QEAAVPR--LR-PH--DNRTMVEIIADHP
Crocodylus porosus MRIPVDPST-SRRFSPPSSSL--------------QP--GKMSEVS----------PAAV---QQQQQQQQQQE------------------------AAAAVPR--LR-PH--DNRTMVEIIADHP
Gekko japonicus MRIPVDPST-SRRFSPPSSSL--------------QP--GKMSEVS----------PAAVAAQQQQQQQQQQQH----------------------QEAAA-PR--LR-PH--DNRTMVEIIADHP
Geotrypetes seraphini MRIPIDPST-SRRFSPPSSSL--------------QPVAGKMSDVS----------PVSV-----------QQE------------------------AAAAVPR--LR-PH--DNRTMVEIIADHP
Latimeria chalumnae MRIPVDPST-SRRFSPPSSSL--------------QPVPGKMTEVS----------PVAG-----------QQE------------------------TAAAVPR--LR-PH--DNRTMVEIIADHP
Lepisosteus oculatus MRIPVDPST-SRRFSPPSNSL--------------QPVTGKMSDVS----------TATG-----------QQE------------------------AAPAVPR--LR-PH--DNRTMVEIIADHP
Danio rerio (Runx2b) MRIPVDPSA-GRRFSPPVS------------------VKMNDVN----------SNAGPQ---------QQQ------------------------DGAVVPR--LR-AQ--ENRSMAEIIADHP
Callorhinchus milii MRIPVDPST-SRRFTPPSTTL---------------PASGKMSDVS----------GMAP-----------HQE--------SGPGVAVAGA-AAAAAAAAALGRSLIR-PH--ENRSMVDIIADHP
Lethenteron camtschaticum (RunxA) MHIPVDAGV-SRRFTPPSTALLHAAHHHHHHHHGSSSSTSKMSSDA----------PPLS-----------HQD-------GALMGVKMRGA-GGGGGGGGGIGGG----AH---DRPMGDVLADHP
Myxine glutinosa (RunxA) MHLPADTGH-TRRFTPPSNTL--------------TP--NKLSDPHHHHHHHHHLHPHHH-----------HHH-------HHQQQHQQQQA-SGAQQGEGALGK--LR-PG---DRPMVEVLADHP
Branchiostoma floridae (runt) MLIPTPSTLDSRRFSPFAD-----------------P--GKMGDP-------------------------HRK-----------------------------------VH-PHFKGDRGLVDALADHP
