# Supplementary material for: Evolution and expansion of the RUNX2 QA repeat corresponds with the emergence of vertebrate complexity
Source: Commun Biol. 2020 Dec 15;3:771. doi: 10.1038/s42003-020-01501-3 (PMC7738678; doi:10.1038/s42003-020-01501-3)
Supplement: Supplementary file 2 — Description of Additional Supplementary Files [file 42003_2020_1501_MOESM2_ESM.pdf]

Description for Additional Supplementary Files

Title: Supplementary data 1

Description: RUNX2 QA repeat composition in fish (Chondrichthyes & Osteichthyes)

Title: Supplementary data 2

Description: RUNX2 QA repeat composition in amphibians (Amphibia)

Title: Supplementary data 3

Description: RUNX2 QA repeat composition in reptiles (Reptilia)

Title: Supplementary data 4

Description: RUNX2 QA repeat composition in birds (Aves)

Title: Supplementary data 5

Description: RUNX2 QA repeat composition in mammals (Mammalia)

Title: Supplementary data 6

Description: Vertebrate simplfied phylogeny Newick Tree
